# Microbubbles-Assisted Ultrasound Triggers the Release of Extracellular Vesicles

**DOI:** 10.3390/ijms18081610

**Published:** 2017-07-25

**Authors:** Yuana Yuana, Linglei Jiang, Bart H. A. Lammertink, Pieter Vader, Roel Deckers, Clemens Bos, Raymond M. Schiffelers, Chrit T. Moonen

**Affiliations:** 1 Imaging Division, University Medical Center Utrecht, Utrecht, 3584 CX, The Netherland; bartlammertink@gmail.com (B.H.A.L.); R.Deckers-2@umcutrecht.nl (R.D.); c.bos@umcutrecht.nl (C.B.); c.moonen@umcutrecht.nl (C.T.M.); 2 Department of Clinical Chemistry and Hematology, University Medical Center Utrecht, Utrecht, 3584 CX, The Netherlands; L.Jiang@umcutrecht.nl (L.J.); pvader@umcutrecht.nl (P.V.); R.Schiffelers@umcutrecht.nl (R.M.S.)

**Keywords:** sonoporation, exocytosis, endocytosis, exosomes, microvesicles, drug delivery

## Abstract

Microbubbles-assisted ultrasound (USMB) has shown promise in improving local drug delivery. The formation of transient membrane pores and endocytosis are reported to be enhanced by USMB, and they contribute to cellular drug uptake. Exocytosis also seems to be linked to endocytosis upon USMB treatment. Based on this rationale, we investigated whether USMB triggers exocytosis resulting in the release of extracellular vesicles (EVs). USMB was performed on a monolayer of head-and-neck cancer cells (FaDu) with clinically approved microbubbles and commonly used ultrasound parameters. At 2, 4, and 24 h, cells and EV-containing conditioned media from USMB and control conditions (untreated cells, cells treated with microbubbles and ultrasound only) were harvested. EVs were measured using flow cytometric immuno-magnetic bead capture assay, immunogold electron microscopy, and western blotting. After USMB, levels of CD9 exposing-EVs significantly increased at 2 and 4 h, whereas levels of CD63 exposing-EVs increased at 2 h. At 24 h, EV levels were comparable to control levels. EVs released after USMB displayed a heterogeneous size distribution profile (30–1200 nm). Typical EV markers CD9, CD63, and alix were enriched in EVs released from USMB-treated FaDu cells. In conclusion, USMB treatment triggers exocytosis leading to the release of EVs from FaDu cells.

## 1. Introduction

Cells from different organisms, including eukaryotes and prokaryotes, have been demonstrated to release vesicles, known as extracellular vesicles (EVs), into the extracellular environment [1,2]. EVs are membrane-enclosed vesicles and comprise different size populations from as large as 2 microns and as small as about 30 nm in diameter [2]. EVs carry protein, nucleic acids, and lipids derived from the parental cells, and have evolved conceptually from being considered as cellular debris to vesicles with a multitude of biological functions including disposal of unwanted substances from cells, wound/plasma membrane repair, emission of signaling and regulatory molecules for cell-cell communication, and stimulation or inhibition of the immune system and antigen presentation [1,3]. Based on the current knowledge, EVs may be classified based on their origin/formation into (1) exosomes, originating from endosomal compartments, (2) microvesicles, originating from outward budding of the plasma membrane, and (3) apoptotic bodies, originating from cells that underwent apoptosis [1,4].

Various drug delivery methods or protocols aimed at targeting cancer cells and tissues have been the subject of investigations for decades [5,6,7]. For the purpose of targeted cancer therapy, sonoporation, a process that involves microbubbles (gas-filled bubbles stabilized by a lipid, protein or polymer shell) interacting with ultrasound (US), has recently gained attention for increasing cellular drug uptake [8,9,10]. In sonoporation (USMB), low-intensity US is commonly used to induce cavitation of microbubbles (MB) which may lead to the formation of transient membrane pores as well as enhanced endocytosis to increase drug uptake by the target cells/tissues [11,12,13,14].

The formation of transient membrane pores induced by USMB has been demonstrated to increase calcium (Ca^2+^) entry to the cells leading to the lysosome exocytosis to the plasma membrane [15,16]. Increased intracellular concentration of Ca^2+^ also has been suggested to be one of the triggers for the release of EVs by cells as part of the action to reseal the disrupted plasma membrane after mechanical lesions (e.g., scrapping, laser) and bacterial infection (e.g., by pore-forming toxins) [17,18]. Based on this rationale, we investigated whether USMB may trigger exocytosis leading to the release of EVs.

In this study, USMB was performed using clinically approved MB at low acoustic pressures on head-and-neck cancer cells (FaDu) in vitro, as published previously [19]. To investigate the release of EVs, conditioned media containing EVs of USMB treated-cells and control cells (untreated cells and cells treated with US or MB only) were collected at different time points (2, 4 and 24 h). The level of EVs carrying tetraspanin proteins such as CD63 (lysosomal-associated membrane protein 3) and CD9 in conditioned media collected from USMB-treated cells were measured by an immune-magnetic bead capture assay coupled to flow cytometry and the results were compared to those of control cells to determine whether the level of EVs increased after USMB. Simultaneously, EVs were quantitated and characterized based on the presence of common EV markers such as CD63, CD9, alix and tsg101 (tumor-susceptibility gene 101), a member of the endosomal sorting complex required for transport, and lactadherin (a milk fat globule-EGF factor 8 protein) to detect surface exposure of the negatively charged phospholipid, phosphatidylserine, either by immunogold electron microscopy or western blotting.

## 2. Results

### 2.1. Conditioned Media of USMB-Treated FaDu Cells Contained Higher Levels of EVs Carrying CD9 and CD63

**Figure 1:**
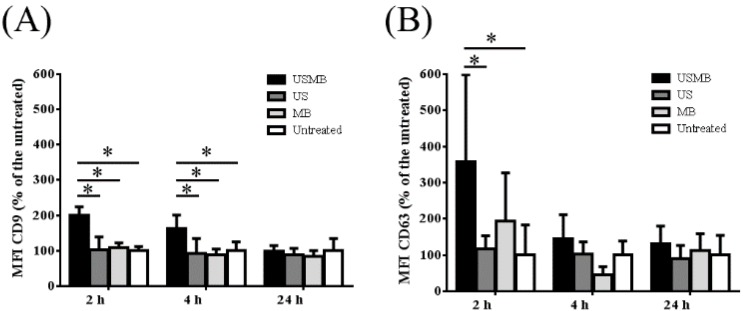
Comparison of the level of mean fluorescence intensities (MFI) of bead-captured extracellular vesicles (EVs) exposing CD9 and CD63 measured using flow cytometry. MFI CD9 and MFI CD63 were corrected from their IgG1 controls. Percentages of MFI CD9 (**A**) and CD63 (**B**) of EVs derived from conditioned media of FaDu cells collected at 2, 4 and 24 h after microbubbles-assisted ultrasound (USMB), ultrasound (US), and microbubbles (MB) treatments were calculated by comparing those with the untreated counterparts. Bars represent mean of three independent experiments + standard deviation. Statistical analysis was performed using two-way ANOVA followed by Tukey’s test. *p* value < 0.05 was considered significant (*).

EVs carrying CD9 and/or CD63 were present in the conditioned media collected at 2, 4 and 24 h after FaDu cells were treated with USMB, US or MB only, and untreated. This is shown by the CD9 and CD63 fluorescent intensities of EVs exposing CD9/CD63 captured by anti-CD9/CD63 antibody coated-magnetic beads. There was no fluorescent signal detected when IgG1 isotype control was used. We found that the CD9 and CD63 mean fluorescence intensities increased over a period of 24 h in all conditioned media containing EVs (USMB, US, MB, and untreated). The level of CD9 mean fluorescent intensities in the conditioned media increased about 2-fold in comparison to the US, MB, and untreated conditions, specifically at 2 and 4 h after USMB (Figure 1A). In the same conditioned media, the level of CD63 fluorescence intensity increased almost 4-fold in the conditioned medium at 2 h after USMB in comparison to the US and untreated conditions (Figure 1B). The increased level of CD63 fluorescence intensity also was found at 4 h after USMB, but this was not significant in comparison to the control conditions (US, MB, and untreated). At 24 h, the levels of CD9 and CD63 fluorescent intensities after USMB were comparable to the control conditions (Figure 1). These results suggest that USMB triggers the release of EVs exposing CD9 and CD63 at 2–4 h after USMB. We did not observe a significant difference in the expression of annexin V and propiodium iodide on FaDu cells harvested at different time points after USMB in comparison to untreated FaDu cells, suggesting that USMB treatment gave no significant percentage of cell death (Figure S1).

### 2.2. EVs Released after USMB Were Heterogeneous in Size and Exposed CD9, CD63, and/or Phosphatidylserine

**Figure 2:**
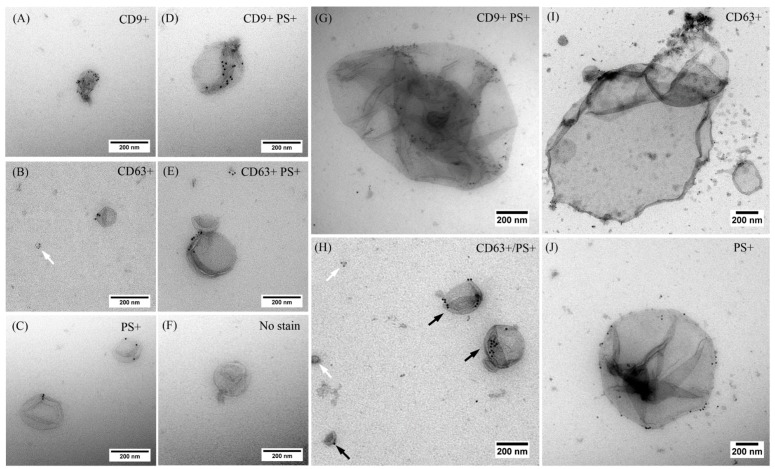
Representative EM images showing immunogold labeling of EVs using anti-CD9 or -CD63 antibody and lactadherin. Both antibodies were coupled with 6-nm gold, whereas lactadherin was coupled with 10-nm gold. EVs were derived from conditioned media of FaDu cells collected at 4 h after microbubbles-assisted ultrasound (USMB) treatment. EVs were stained positive by anti-CD9 antibody (**A**), anti-CD63 antibody (**B**,**H** white arrows, and **I**), or lactadherin, a surface marker of the negatively charged phospholipid, phosphatidylserine (PS+; **C**,**H** black arrows, and **J**) only. Some were double stained by anti-CD9 antibody and lactadherin (**D**,**G**) or anti-CD63 antibody and lactadherin (**E**). EVs without gold label were also detected (**F**). Large EVs with an apparent diameter of 500–1200 nm were also detected (**G**,**I**,**J**).

Following our findings on the increased levels of CD9 and CD63 exposing EVs in early time points after USMB, we performed immunogold staining followed by EM imaging to visualize and characterize individual EVs present in the conditioned media collected at 4 h after USMB and their control conditions (US, MB, and untreated) based on the presence of CD9, CD63, and/or a negatively charged phospholipid, phosphatidylserine (shown by the positive staining of lactadherin) on their surface. Double staining using anti-CD9 antibody and lactadherin or anti-CD63 antibody and lactadherin was performed on EVs followed by EM imaging. Representative EM images of EVs detected in the conditioned media of USMB condition are shown in Figure 2. Clearly, EVs were heterogeneous in size and exposed CD9, CD63, and/or phosphatidylserine on their surface. EVs with an apparent diameter of 30–1200 nm were visible (Figure 2). We detected EVs stained only by anti-CD9 or CD63 antibody, or lactadherin, whereas others were double stained by anti-CD9 antibody and lactadherin or anti-CD63 antibody and lactadherin (Figure 2A–E,G–J). Also EVs without gold label were present in the conditioned medium which was double stained by anti-CD9 antibody and lactadherin (Figure 2F). There was no staining on EVs when IgG1 isotype control was used. These results indicated that EVs released after USMB were comprised of heterogeneous vesicles exposing CD9, CD63, and/or phosphatidylserine.

**Figure 3:**
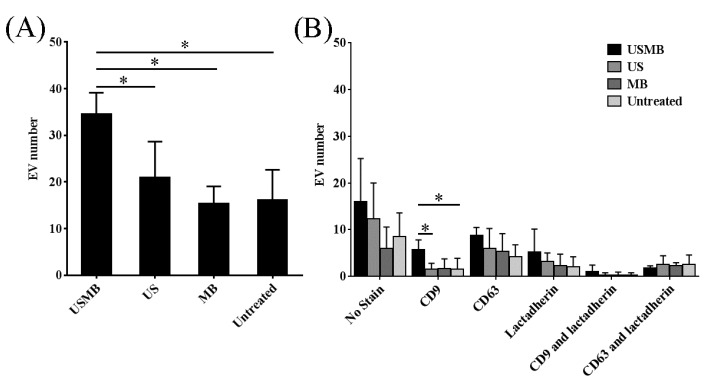
Quantification of EVs detected using EM in conditioned media collected at 4 h from cells after microbubbles-assisted ultrasound (USMB), ultrasound (US), or microbubbles (MB) treatments, and from untreated cells. Ten images per grid recorded at 60,000 × magnification were used for analysis. Total number of EVs from USMB treatment was significantly higher than those from US or MB treatment or untreated (**A**). Subpopulations of single, double stained, or unstained EVs were quantified (**B**). EV number represents the mean of four independent experiments + standard deviation. Statistical analysis was performed using one-way ANOVA followed by Tukey’s test. *p* value < 0.05 is considered significant (*).

We quantified the total number of EVs and number of EVs unstained and stained by anti-CD9, anti-CD63, and/or lactadherin which were present in the conditioned media collected at 4 h (Figure 3). Noticeably, total number of EVs detected in the conditioned media collected after USMB increased about 2-fold compared to those detected in the conditioned media of control conditions (US, MB, and untreated) (Figure 3A). Particularly, the number of EVs exposing CD9 increased 4-fold in USMB condition in comparison to the US and untreated conditions (*p* value < 0.05, Figure 3B), corresponding to the results obtained from flow cytometric immuno-magnetic bead capture assay (Figure 1A).

**Figure 4:**
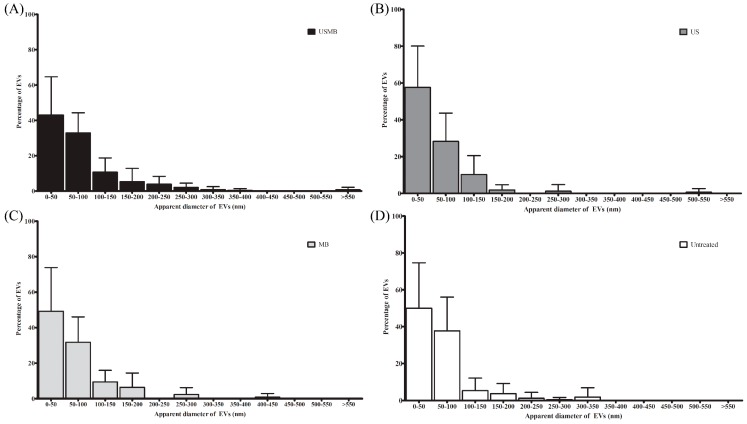
Size distribution of EVs detected in conditioned media collected at 4 h from cells after microbubbles-assisted ultrasound (USMB, **A**), ultrasound (US, **B**), or microbubbles (MB, **C**) treatments, and from untreated cells (**D**). Surface area of EVs was measured using Image J [20] to calculate the apparent diameter of EV (nm). Bin size is 50 nm. EVs were detected from 10 images per grid (60,000× magnification) derived from four independent experiments. Percentage of EVs represents the percentage of mean of EVs at certain size range + standard deviation.

EVs detected in the conditioned media of USMB, US, MB, and untreated conditions were heterogeneous with an apparent diameter of 30–1200 nm (Figure 2 and Figure 4). In all conditioned media (USMB, US, MB, and untreated), EVs with an apparent diameter size below 200 nm, often considered to represent the EV population of exosomes, were most abundant (Figure 4). CD63, but not CD9 and lactadherin, seemed to be overrepresented in the population of these small-sized EVs (Figure S2). EVs larger than 200 nm (8 ± 1%) of the total EV population were mainly observed in the conditioned media of USMB condition (Figure 4A), whereas in the controls (US, MB, and untreated), most EVs (97 ± 1%) were below 200 nm (Figure 4B–D).

### 2.3. Alix, CD9, CD63, and Calnexin Were Present in EV Lysates after USMB

**Figure 5:**
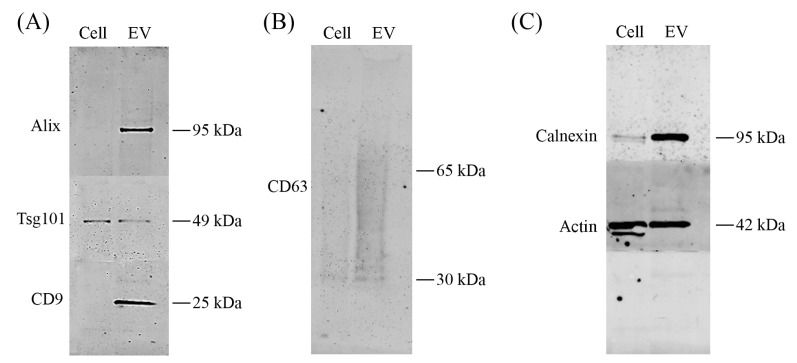
Western blotting on total cell and EV lysates prepared from FaDu cells and their supernatant collected at 4 h after microbubbles-assisted ultrasound (USMB). For western blotting, equal protein amounts of cell and EV lysates were loaded (4.5 µg of total proteins). Detection of the proteins was by using antibodies alix, tsg101, CD9, CD63, calnexin, and actin. Presence of alix (95 kDa), tsg101 (49 kDa), and CD9 (25 kDa) are shown (**A**). CD63 is shown as a broad band between 30–65 kDa (**B**). Calnexin (95 kDa) and actin (42 kDa) are present in both cell and EV lysates (**C**).

To investigate whether USMB treated-FaDu cells sorted specific proteins into EVs, we assessed protein content of FaDu cells and their EVs at 4 h after USMB using western blotting. Detection antibodies for alix, tsg101, CD9, CD63, calnexin, and actin were used. EVs released after USMB expressed typical EV markers alix, tsg101, CD9, and CD63 shown by specific protein bands on the membrane (Figure 5A,B). Actin was found in both lysates of USMB-treated cells and their EVs (Figure 5C). Especially, alix, CD9, CD63, and calnexin were enriched in EV lysates compared to USMB treated-FaDu cells from which EVs were derived (Figure 5A–C). The levels of alix, CD9, tsg101, actin, and calnexin detected in lysates of USMB-treated cells were comparable to those of untreated cells (Figure S3). In addition, alix, tsg101, CD9, and CD63 in EV lysates derived from untreated cells were below the detection limit. Taken together, FaDu cells released EVs after USMB and sorted specific proteins into these EVs. Besides typical EV markers, calnexin was overexpressed indicating that not only exosomes were present in this EV lysate after USMB, but also other types of vesicles as shown from results obtained from the EM analysis (Figure 2, Figure 3 and Figure 4).

## 3. Discussion

In the current study, we report that USMB triggers the release of EVs from FaDu cells. We found that levels of CD9 exposing-EVs significantly increased at 2 and 4 h, whereas levels of CD63 exposing-EVs increased at 2 h after USMB. At 24 h, levels of CD9 and CD63 exposing-EVs after USMB were comparable to the control levels (US, MB, and untreated). An increased number of EVs also was detected in the conditioned medium at 4 h after USMB in comparison to conditioned medium of control FaDu cells (US, MB, and untreated). EVs triggered by USMB carried typical EV markers such as CD9, CD63, alix, and tsg 101. Proteins CD9, CD63, alix, and calnexin present in the lysate of USMB treated-FaDu cells were enriched in their EVs indicating that USMB treated-FaDu cells sorted these proteins through EVs.

Calnexin is an endoplasmatic reticulum protein and is generally accepted to be absent from exosome preparation [21,22,23]. The fact that we did find calnexin in our EV preparations (Figure 5C) confirms our earlier findings that our EV preparations contained not only exosomes but also other subtypes of EVs (e.g., microvesicles or apoptotic bodies) that can carry calnexin (Figure 2 and Figure 4). Our intention was to study total EV population after USMB. Therefore, we did not extensively purify exosomes using differential (ultra) centrifugation as by using extensive purification steps we may lose EV subpopulation(s) and cause aggregates of EVs, which was not desirable for EV quantification [24].

The CD9 and CD63 mean fluorescence intensities increased over a period of 24 h in all conditioned media containing EVs (USMB, US, MB, and untreated). This indicates that the EV concentrations at 2 h after USMB application may be lower than those at 4 and 24 h after USMB application. Since measurements using immunogold EM and western blotting are concentration dependent, it is desirable to measure EVs at higher concentration. Therefore, we measured EVs at 4 h-time point instead of at 2 h-time point after USMB to optimally analyze the data.

In the current study, we used FaDu cells and similar experimental settings as described by Lammertink et al. [19]. Using these settings, the measured duration of cell membrane permeability after US exposure was expected in the order of 1–3 h after USMB [25]. We observed the induction of EVs release by FaDu cells in a similar time period, which may be connected to a transient increase in membrane permeability. Further experiments, which are beyond the scope of the present study, are needed to investigate whether the generation of EVs and their protein content are influenced by different types of cavitation or different concentrations of MB.

It has been suggested that intracellular vesicles exocytosed and accumulated at sites of mechanically wounded plasma membrane and extracellular shedding of wounded plasma membrane promotes wound repair [26]. Thus, our findings imply that a rapid release of EVs may also be part of processes of plasma membrane repair after USMB. Whether the EV release is connected to the Ca^2+^ influx due to transient plasma membrane modification by USMB is one of the questions that remains to be addressed. Lastly, further understanding of the function of USMB-triggered EVs may be important for improved drug delivery using USMB in the near future.

## 4. Materials and Methods 

### 4.1. Cell Culture

Human pharyngeal squamous carcinoma cells (FaDu) (ATCC^®^ HTB-43™, LGC Standards GmbH, Wesel, Germany) were cultured in high glucose Dulbecco’s Modified Eagle Medium (DMEM) (Sigma-Aldrich, Zwijndrecht, The Netherlands), supplemented with 1% Non-Essential Amino Acids (NAA) (Sigma-Aldrich, Zwijndrecht, The Netherlands) and 10% fetal bovine serum (FBS) (Sigma-Aldrich). Cells were maintained in standard cell culture flasks in a humidified incubator at 37 °C and 5% CO_2_.

### 4.2. Cell Preparation for USMB

For USMB experiments, cells were prepared according to our previously reported method with slight modifications [19]. Briefly, 1 × 10^6^ cells were seeded in cell culture chambers (CLINIcell^®^ 175 μm thick polycarbonate walls, 25 cm^2^) (Mabio, Tourcoing, France) coated with 0.01% poly-l-lysine (molecular weight 70,000–150,000, Sigma-Aldrich), 1 day prior to US exposure. SonoVue™ (Bracco, Milan, Italy) lipid shelled MB, encapsulating sulfur hexafluoride gas (SF6) were used in the USMB experiments [27]. MB were prepared according to the manufacturer’s guidelines, yielding a mean diameter of 2.5 μm and a concentration between 1 and 5 × 10^8^ MB/mL. Prior to USMB, MB (700 μL) were mixed with 9.5 mL medium consisting of OptiMEM (Thermo Fisher Scientific, Rockford, IL, USA), 1% EV depleted-FBS (Thermo Fisher Scientific, Rockford, IL, USA), and 1% antibiotic-antimycotic solution (Sigma-Aldrich). This microbubble-containing medium (final concentration of 0.7–3.5 × 10^8^ MB) was then injected and the cell culture chamber (containing about 1.45 × 10^6^ attached cells) was placed upside down, allowing the MB to rise by floatation towards the cells, ensuring close contact between cells and MB.

### 4.3. USMB Treatment

**Figure 6:**
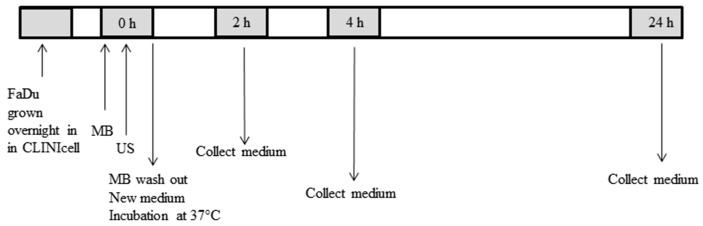
Timeline of microbubbles-assisted ultrasound (USMB) experiment of FaDu cells. First, medium containing microbubbles (MB) were added into the FaDu cells grown overnight in the cell culture chamber, and then ultrasound (US) was applied. Medium was directly removed after USMB and cells were washed once. Cells received new medium and incubated for 2, 4, and 24 h. At these time points, cells and conditioned medium were collected.

FaDu cells grown in a cell culture chamber received USMB and the others received US or MB only, or without treatment. The US setup used was similar to the one that was previously described [19]. In short, a 1.5 MHz sinusoidal signal was created by an arbitrary waveform generator (Agilent Technologies, Santa Clara, CA, USA), which was triggered by an oscilloscope (Agilent Technologies) to generate a pulsed waveform (10% duty cycle, 1 kHz pulse repetition frequency, and 100 μs pulse duration). The wave was amplified (KMP Electronics, Bédoin, France) and sent to a piezoelectric unfocused single element transducer (Precision Acoustics, Dorchester, UK) with a diameter of 20 mm. The transducer was placed on the bottom of a water tank, 8 cm below a cell culture chamber frame. The water surface was another 12 cm above the frame and heated to 37 °C. When exposing cells to US, the cell culture chamber was mounted in the frame and moved over the US beam for 80 s to expose the whole cell culture chamber surface, which resulted in a total exposure of approximately 5 s for each area of the cell culture chamber [19]. Cells were exposed to 845 kPa acoustic pressure, as was calibrated using a 125 μm glass fiber hydrophone (Precision Acoustics). The USMB timeline is presented schematically in Figure 6.

Directly after exposure to US, the microbubble-containing medium in the cell culture chamber was removed and cells were washed once with phosphate-buffered saline (PBS). Afterwards, new medium (OptiMEM containing 1% exosome depleted-FBS and 1% antibiotic-antimycotic solution) was injected in the cell culture chamber and cells were incubated for 2, 4 and 24 h. At these time points, conditioned medium was harvested and centrifuged at 300× *g* for 10 min at room temperature (RT) to remove detached cells and large cellular debris. Supernatant was collected, snap-frozen in liquid nitrogen, and stored at −80 °C for subsequent measurements. Finally, attached cells in the cell culture chamber were detached using trypsin- ethylenediaminetetraacetic acid (EDTA) solution (Sigma-Aldrich). After medium (DMEM supplemented with 1% NAA and 10% FBS) was added, cells were pelleted by centrifuging at 300× *g* for 5 min at RT. The cell pellet was resuspended with 1 mL medium and then cells were stained with tryphan blue (Biorad, Veenendaal, The Netherlands) before counting using a Biorad TC20 automated cell counter. Of note, there was >80% cell viability upon exposure to USMB treatment. Additionally, the Dead Cell Apoptosis Kit with annexin V fluorescein isothiocyanate (FITC) and propiodium iodide (Thermo Fisher Scientific) was used to detect apoptosis/death cells in the USMB experiments.

### 4.4. Flow Cytometric Immuno-Magnetic Bead Capture Assay

EVs were directly assessed in the conditioned medium without any isolation or purification steps to avoid EV aggregate formation or loss of subpopulations of EVs as reported elsewhere [24,28]. ExoCap magnetic capture beads CD9 and CD63 from JSR Life Sciences (Leuven, Belgium) were used according to the manufacturer’s instructions. Briefly, ExoCap magnetic capture beads CD9 or CD63 were pipetted to a 96-well round-bottom plate (end concentration of beads in each well was 6 × 10^4^ beads) and incubated with 70 μL samples overnight at 4 °C with gentle agitation. On the next day, the plate was placed on the magnetic plate separator for 2–3 min and the supernatant was removed. Beads were washed by adding 100 μL 0.5% bovine serum albumin (BSA)-containing PBS (0.45 μm filtered) to each well following a gently shaking of the plate for 5 min at RT. After placing the plate on top of the magnetic plate separator, the supernatant was removed. Diluted anti-CD9, anti-CD63, or the corresponding IgG1 isotype control coupled to Alexa Fluor 647 (100 µL) were added to the respective wells (see Table S1 for the dilutions). This mixture was incubated at RT in the dark for 2 h. Lastly, the plate was again placed on top of the magnetic plate separator and the antibody/isotype control solution was removed. The plate was washed once with 0.5% BSA-containing PBS. Samples containing EVs attached to the beads were re-suspended in 250 μL PBS (0.45 µm filtered) and measured using FACS canto-II (BD biosciences, San Jose, CA, USA). Three independent experiments were performed and from each experiment, samples were measured in duplicate to assess the levels of CD9 and CD63.

### 4.5. Immunogold Electron Microscopy

A thin layer of carbon on the 200 mesh formvar-carbon coated copper grids (Electron Microscopy Sciences, Hatfield, PA, USA) was evaporated using a carbon-vacuum evaporator according to the manufacturer’s instructions (Edwards Auto 306, West Sussex, UK) just before sample application. EVs were not concentrated using high-speed or ultra-centrifugation prior to electron microscopy (EM) analysis. Instead, grids were floated directly on top of 10 μL of conditioned medium containing EVs and incubated for 7 min at RT. The grids were washed three times using PBS (0.22 µm filtered). Next, the grids were incubated in a blocking solution (Aurion, Wageningen, The Netherlands) for 30 min at RT followed by washing three times with 0.1% BSA-c (Aurion). For double immuno-labeling, the grids were incubated overnight at 4 °C in a mixture of 5 µL of mouse anti-human primary antibody (anti-CD9 or anti-CD63 antibody) and 5 µL lactadherin FITC. For negative controls, mouse IgG1 isotype control and PBS were used. Unbound antibodies were removed from the grids in six washing steps by placing the grids on top of 0.1% BSA-c solution. Afterwards, the grids were incubated with 10 µL goat anti mouse IgG secondary antibody labeled with 6-nm gold particles (Aurion) for 1 h at RT. Grids were washed six times with 0.1% BSA-c before incubation with 10 µL mouse anti-FITC labeled with 10-nm gold particles for 1 h at RT. Dilutions of the antibodies, isotype control, and immunogold particles can be found in Table S1. Grids were washed again six times with 0.1% BSA-c, followed by three times washing with PBS. To fix the labeled sample, the grid was incubated for 5 min with 2% glutaraldehyde and washed six times with ultrapure water. Next, the grids were transferred to 0.4% uranyl acetate in 2% methyl cellulose and incubated for 10 min. Excess solution on the grids was removed by blotting the grid at 45° angle once from the side of the grid with filter paper. Grids were imaged using a Tecnai 12 electron microscope operated at 80 kV. For quantification of vesicles, 10 images per grid were recorded at 60,000× magnification and the surface area of each vesicle was measured using ImageJ [20] to calculate the vesicle diameter. The presence of 6-nm gold particle on the EV surface indicated the presence of CD9 or CD63, whereas the presence of 10-nm gold particle indicated the presence of lactadherin staining on the EV surface. Four independent experiments were performed to quantify EVs from each conditioned medium (USMB, US, MB, and untreated).Quantification per condition was performed using four grids.

### 4.6. Western Blotting

To isolate EVs for analysis by western blotting, conditioned media were centrifuged at 110,000× *g* for 70 min, 4 °C using a fixed-angle type 50.2 Ti Rotor (Beckman Coulter, Indianapolis, IN, USA). For analysis of the protein content of cells and EVs, cells and EV pellets were lysed in RIPA buffer (Sigma-Aldrich) supplemented with protease inhibitor cocktail (Thermo Fisher Scientific, Rockford, IL, USA) for 30 min on ice with vortexing at 10 min intervals. These cell and EVs extracts were centrifuged at 12,000× *g* for 15 min at 4 °C and the clear lysates were collected. Protein concentration of each lysate was determined using a Micro BCA Protein Assay Kit (Thermo Fisher Scientific). Equal amounts of proteins from cell and EV lysates were used for western blotting. These lysates were mixed with sample buffer containing dithiothreitol (except for CD63 protein detection), heated to 100 °C for 10 min, and loaded into 4–12% bis-tris polyacrylamide gels (Thermo Fisher Scientific). The electrophoresis was done at 165 V for 45 min. Proteins were blotted on Immobilon-FL polyvinylidene difluoride (PVDF) membrane (Millipore, MA, USA). Blotting was done at 125 V for 60 min. Afterwards, the PVDF membrane was blocked overnight at 4 °C in a mixture of Odyssey blocking buffer (LI-COR Biosciences, Leusden, the Netherlands) and tris-buffered saline (TBS) in a ratio 1:1. Subsequently, membrane was probed with primary antibodies (Table S1) in 50% volume/volume Odyssey blocking buffer in TBS with 0.1% Tween20 (TBS-T) at RT for 2 h. The membrane was washed with 1% TBS-T and incubated with secondary antibodies (Table S1) at RT for 1 h. Finally, the membrane was washed and protein bands were visualized using an Odyssey infrared imager (LI-COR Biosciences) at a wavelength of 700 nm.

### 4.7. Statistical Analysis

Statistical analysis was performed using GraphPad Prism 6 (La Jolla, CA, USA). ANOVA (one or two-way) followed by Tukey’s multiple comparison tests were used to analyze and define significant differences between samples after USMB and controls (US, MB, or untreated) were executed at specific time points (2, 4 and 24 h). Differences were considered significant when *p*-values were < 0.05.

## Supplementary Materials

Supplementary materials can be found at www.mdpi.com/1422-0067/18/8/1610/s1.
