# Supplementary material for: Microbubbles-Assisted Ultrasound Triggers the Release of Extracellular Vesicles
Source: Int J Mol Sci. 2017 Jul 25;18(8):1610. doi: 10.3390/ijms18081610 (PMC5578002; doi:10.3390/ijms18081610)
Supplement: Supplementary file 1 [file ijms-18-01610-s001.pdf]

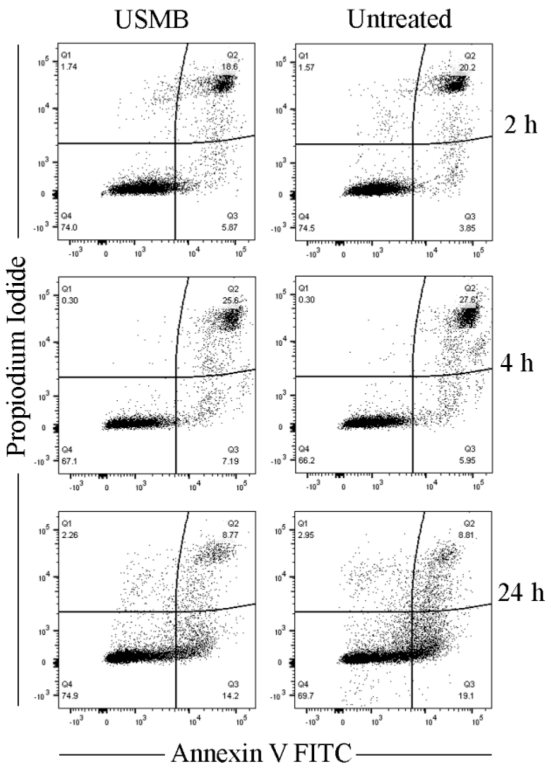

Figure S1. Double staining of annexin V fluorescein isothiocyanate (FITC) and propiodium iodide on microbubbles-assisted ultrasound (USMB)-treated and untreated cells harvested at 2, 4, and 24 h.

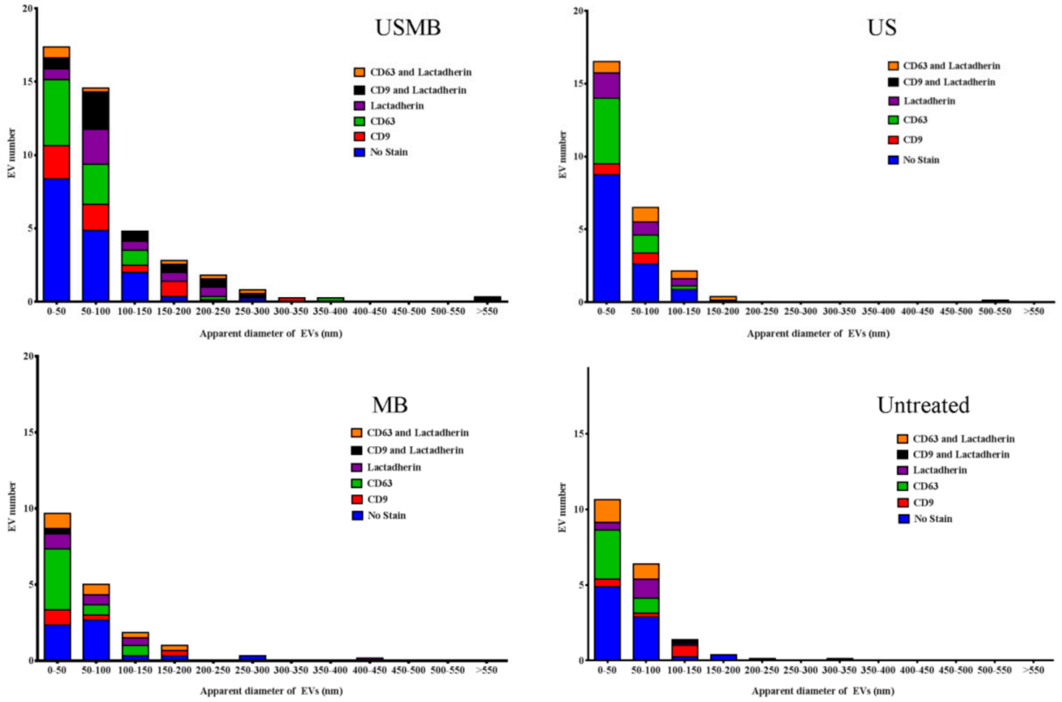

Figure S2. Size distribution of EVs exposing CD9, CD63, and/or lactadherin. These EVs were derived from the conditioned media collected at 4 h after microbubbles-assisted ultrasound (USMB), ultrasound (US) or microbubbles (MB) treatment only, and untreated. Surface area of EVs was

measured using ImageJ [20] to calculate the apparent diameter of EV (nm). Bin size is 50 nm. EV numbers were calculated from the mean of four independent experiments.

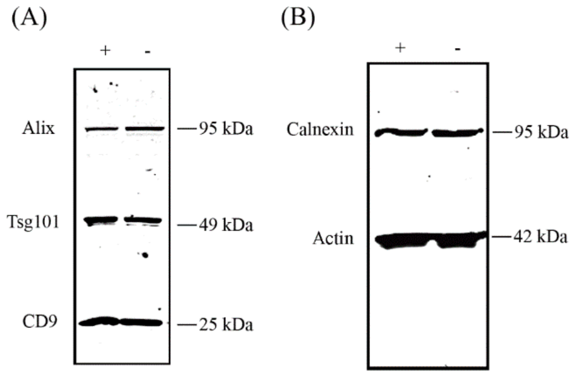

Figure S3. Western blotting on total cell lysates prepared from microbubbles-assisted ultrasound (USMB)-treated (+) and untreated (−) FaDu cells collected at 4 h-time point. For western blotting, equal protein amounts of cell lysates were loaded (8.8 μg of total proteins). Detection of the proteins was by using antibodies alix, tsg101, CD9, calnexin, and actin. Presence of alix (95 kDa), tsg101 (49 kDa), and CD9 (25 kDa) are shown (**A**). Calnexin (95 kDa) and actin (42 kDa) are present in both cell and EV lysates (**B**).

Table S1. Antibodies used in bead capture assay, western blotting, and immunogold electron microscopy.

Name	Clone	Dilution	Manufacturer	Application
Alexa Fluor 647 mouse anti-human CD9 antibody	MEM-61	1:200	EXBIO Antibodies, Vestec, Czech Republic	Bead capture assay
Alexa Fluor 647 mouse anti-human CD63	H5C6	1:200	BD Pharmingen, San Jose, CA, USA	Bead capture assay
Alexa Fluor 647 mouse IgG1κ isotype control	MOPC-21	1:200	BD Pharmingen	Bead capture assay
Mouse anti-human alix antibody	3A9	1:1000	Abcam, Cambridge, UK	Western blotting
Mouse anti-human actin	JLA20	1:1000	Merck Millipore, Amsterdam, The Netherlands	Western blotting
Rabbit anti-human histone 2B	Polyclonal	1:1000	Abcam	Western blotting
Rabbit anti-human CD9	EPR2949	1:2000	Abcam	Western blotting
Rabbit anti-human tsg101	Polyclonal	1:1000	Abcam	Western blotting
Rabbit anti-human Calnexin antibody	Polyclonal	1:1000	GeneTex, Irvine, CA, USA	Western blotting
Mouse anti-human CD63 antibody	MEM-259	1:600, 1:10	Abcam	Western blotting, electron microscopy
Alexa 680 goat anti-mouse		1:10,000	Thermo Fisher Scientific, Rockford, IL, USA	Secondary antibody, western blotting
Alexa 680 goat anti-rabbit (biotin)		1:10,000	Thermo Fisher Scientific	Secondary antibody, western blotting
Mouse anti-human CD9	M-L13	1:50	BD Pharmingen	Primary antibody, electron microscopy
Lactadherin FITC		1:20	Haematologic Technologies Inc., Essex Junction, VT, USA	Electron microscopy
Mouse IgG1 isotype control	X40	1:50	BD Biosciences, San Jose, CA, USA	Electron microscopy
Goat anti-mouse IgG 6 nm		1:50	Aurion, Wageningen, The Netherlands	Electron microscopy
Mouse anti-FITC 10 nm		1:20	Aurion	Electron microscopy
